# Bending Fatigue Behavior of 316L Stainless Steel up to Very High Cycle Fatigue Regime

**DOI:** 10.3390/ma13214820

**Published:** 2020-10-28

**Authors:** Yongtao Hu, Yao Chen, Chao He, Yongjie Liu, Qingyuan Wang, Chong Wang

**Affiliations:** 1 Failure Mechanics and Engineering Disaster Prevention and Mitigation Key Laboratory of Sichuan Province, Sichuan University, Chengdu 610207, China; yongthu@163.com (Y.H.); chenyao1101@126.com (Y.C.); hechao@scu.edu.cn (C.H.); liuyongjie@scu.edu.cn (Y.L.); 2 MOE Key Laboratory of Deep Earth Science and Engineering, College of Architecture and Environment, Sichuan University, Chengdu 610065, China; 3 School of Architecture and Civil Engineering, Chengdu University, Chengdu 610106, China

**Keywords:** 316L SS, VHCF, bending fatigue, fatigue mechanism, microstructure

## Abstract

Effect of microstructure on the crack initiation and early propagation mechanism in the very high cycle fatigue (VHCF) regime was studied in 316L stainless steel (316L SS) by atomic force microscope (AFM) and electron back scattered diffraction (EBSD). The results show that small fatigue cracks initiate from the slip band near the grain boundaries (GBs) or the twin boundaries (TBs). Early crack propagation along or cross the slip band is strongly influenced by the local microstructure such as grain size, orientation, and boundary. Besides, the gathered slip bands (SBs) are presented side by side with the damage grains of the run-out specimen. Finally, it is found that dislocations can either pass through the TBs, or be arrested at the TBs.

## 1. Introduction

316L SS is widely used as the nuclear engineering structural material, such as the support plate, primary coolant pipe, and main coolant pump in a pressurized reactor, owing to its better welding performance [1], low radiation sensitivity [2], excellent corrosion and oxidation resistance [3], et cetera. These components are often subjected to high frequency and low-stress amplitude, especially because of flow-induced vibration, so fatigue failure usually occurs.

The failure mechanism of bending fatigue of long pipe in the reactor due to flow-induced vibration is studied in the present work, which belongs to the very high cycle fatigue (VHCF) regime. Due to the diversity of fatigue test loading, the existing research is still focused on the material’s fatigue performance, and the mechanism of fatigue crack initiation and early propagation is not fully understood [4,5,6]. Moreover, the research on the symmetrical bending of metal materials by ultrasonic fatigue technology is rare. There is only one research on the bending vibration fatigue performance of TC17 titanium alloy [7].

Due to the complexity of the microstructure (such as grain size, grain orientation, twins, etc.) of metal materials, the study of fatigue crack initiation mechanism is challenging. Even when the fatigue loading is macro elastic, the inhomogeneity of adjacent grains in the material will lead to permanent (inelastic) microstructure deformation in the local region, and the stress concentration caused by the accumulation of such deformation plays a leading role in the early initiation of fatigue cracks [8,9,10,11,12,13,14]. For polycrystalline materials such as 316L SS that do not contain obvious internal defects, the internal atoms will undergo dislocation slip motion under the action of shear stress, and then many slip bands will be formed on the free surface of the specimen, so intrusion and extrusion are often accompanied between the matrix and the slip band. The vacuum mode annihilation of dislocations moving forward and backward in the slip band can be regarded as infinitesimal crack propagation [15,16]. The research on the formation and development of slip bands can provide more details for the mechanism study of fatigue crack initiation. The slip band with the associated extrusion and intrusion has unique geometry and its formation process will cause the change of surface roughness. An atomic force microscope (AFM) can supplement the optical measurement of the deformation of the specimen from macro-level to the micro-level, and can also quantitatively detect the fatigue damage changes of the specimen surface. For example, AFM was used to slow crack propagation mechanisms in a glassy polymer [17], obtain the quantitative data of the change process of 3D morphology [18], and study the deformation mechanics of intrusions and extrusions of the slip bands [19]. Its accuracy depends on the geometric dimensions of marks and the AFM probes.

Under low-stress amplitude, the accumulation of irreversible plastic deformation not only leads to fatigue failure at the persistent slip band. Because the slip band can be blocked by barriers such as grain boundaries, dislocations pile up at this place, causing local deformation and incompatibility at the grain boundary, resulting in the grain boundary becoming an important position for fatigue crack initiation [20,21,22,23]. For the single-phase material Alloy 690, the strain localization due to the accumulation of dislocations at the grain boundary or near the grain boundary will eventually become the source of fatigue cracks in the VHCF regime [24]. Besides, when the barrier caused by microstructures such as grain boundaries is strong enough, the growth of small fatigue cracks will be suppressed, so that the material does not experience fatigue failure. For example, in the cyclic deformation structure of 316L austenitic stainless steel, it is observed that the growth of ultra-high cycle fatigue cracks is hindered by coherent twins [25]. For another example, the phase boundary of austenite-ferrite dual-phase steel is an effective barrier to prevent slip transfer [26,27].

However, whether the barrier can achieve infinite life depends largely on the interaction of the local plastic deformation process, small fatigue crack nucleation, and their subsequent interaction with the microstructure of surrounding materials [28]. Therefore, the mechanism of fatigue crack initiation and propagation of 316L SS at low-stress amplitude (VHCF) needs to be studied in detail.

## 2. Experiment Procedures

### 2.1. Materials and Specimens

An austenitic stainless steel was used in this study. Its chemical composition is C: 0.03; Si: 0.566; Mn: 1.138; P: 0.023; S: 0.0016; Ni: 16.878; Cr: 10.21; Cu: 0.014; N: 0.0371; Mo:2.045; and Fe: balance. As-received materials were heat-treated by solution-annealing at 1050 ℃ for 30 min and followed by water quenching (referred to ST after this). Three standard tensile specimens were prepared after the heat-treatment, and tensile testing was carried out on a 100kN tensile test machine (SHIMADZU, Kyoto, Japan) with a constant strain rate of ${5\times 10}^{-4}\;s^{-1}$. The hardness testing was performed with a load of 0.5 kg. The mechanical properties of ST 316L SS (TISCO, Taiyuan, China) are indicated in Table 1.

**Figure 1:**
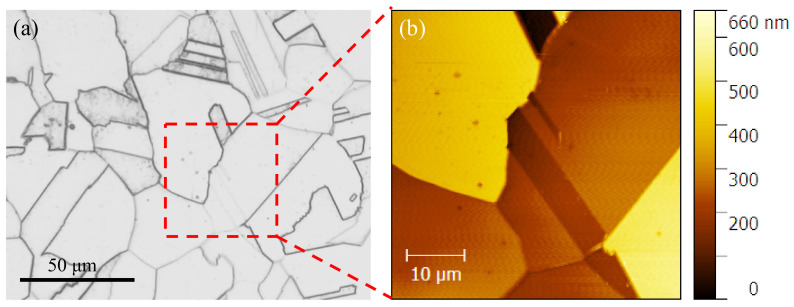
Microstructure of ST 316L: (**a**) OM image and (**b**) AFM image.

Metallographic samples were prepared following the standard grinding and polishing procedure. The ratio of metallographic corrosion solution for 316L SS is FeCl_3_:HCl:H_2_O = 1:1:3. The etching was conducted by immersing the sample in the above solution for 5 to 10 s, and then taking it out immediately and washing it with clean water. Figure 1a shows the microstructure of ST 316L SS in the optical microscope (OM), in which the average grain size is about 50 μm according standard process suggested by ASTM E112-10. The AFM image (Figure 1b) shows many annealed twins distributed on the austenite grains; the average roughness of this area is 0.3 μm.

**Figure 2:**
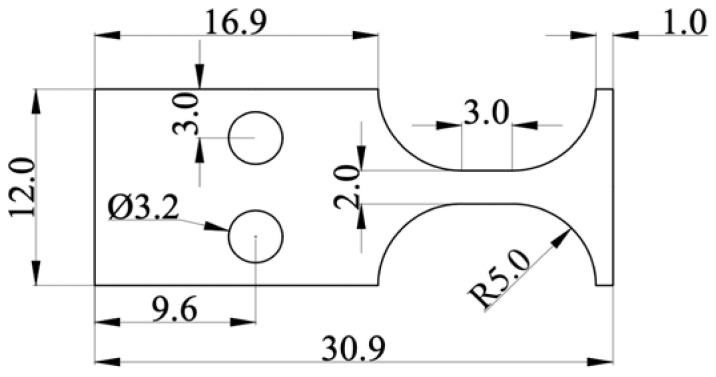
Sketch of ultrasonic symmetrical bending fatigue specimen (dimensions in mm).

The type of specimen selected for the ultrasonic symmetrical bending fatigue test is a plate specimen with a thickness of 1.5 mm. Figure 2 shows the detailed dimensions of it. Each specimen’s surface was polished with #800, #1200, and #1500 grit sandpaper in turn, and then was electrolytically polished by 5% perchloric acid alcohol solution for reducing the effects of the resistant stress.

### 2.2. Ultrasonic Fatigue Test

Similar to conventional tension-compression fatigue tests, symmetrical bending fatigue tests were conducted using an ultrasonic fatigue testing system (Shimadzu, USF-2000, Kyoto, Japan). The excitation signal generated by the ultrasonic generator was loaded on the specimen by transducer and amplifier. The symmetrical bending fatigue specimen was attached to the ultrasonic horn with an attachment system, special spacers with bolts, to constitute the resonance system working at 20 kHz, thereby causing symmetrical bending fatigue (ratio of stress R = −1).

When the resonance frequency was lower than 19.5 kHz, fatigue failure occurred because the resonance system’s frequency gradually decreased as the fatigue damage of the specimen accumulated. In addition, when the number of loading cycles exceeded 10^9^ cycles, the fatigue test was stopped, and the specimen was regarded as a no failure one.

Cooled compressed air and intermittent loading were used during the ultrasonic fatigue test to reduce the thermal effect. The loading and arresting time were selected as 110 ms and 800 ms, respectively.

## 3. Results

### 3.1. Test Results and Fractography

**Figure 3:**
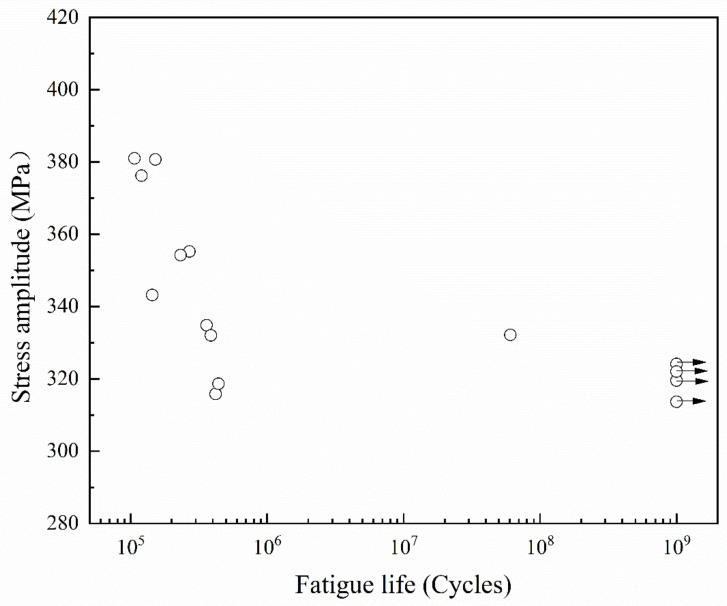
Fatigue results of ST 316L SS.

The test results of ST 316L SS in the very high cycle fatigue regime are shown in Figure 3. The hollow circle represents the fatigue failure specimens, and the hollow circle with an arrow indicates specimens passing the fatigue test at 10^9^ cycles without failure. It is shown that the fatigue life gradually increases with decreasing stress amplitude before 10^6^ cycles. Another important feature is that the stress amplitude, which hardly broke between 10^6^ and 10^9^ cycles, termed as fatigue stress limit here, has been determined as 320 MPa. According to the literature, it has been reported that low-strength steels like 316L SS usually have similar fatigue limits in the VHCF regime [29,30].

**Figure 4:**
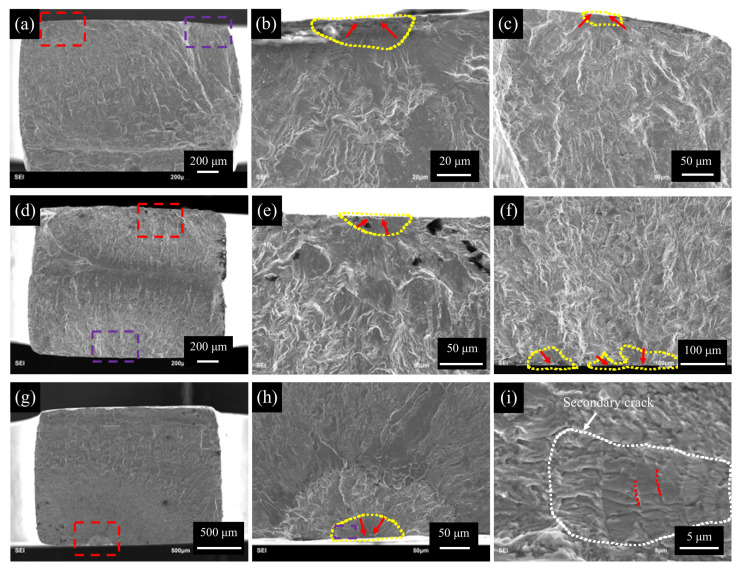
Typical fracture morphologies of surface initiations: (**a**–**c**) σ_0_ = 355 MPa, N_f_ = $2.09\times 10^{5}$ cycles, (**d**–**f**) σ_0_ = 316 MPa, N_f_ = $4.2\times 10^{5}$ cycles, (**g**–**i**) σ_0_ = 332 MPa, N_f_ = $6.09\times 10^{7}$ cycles.

SEM examines the fracture surfaces of all failed specimens. As shown in Figure 4, there are three typical ultra-high cycle bending fatigue fracture morphologies, corresponding to high-cycle (i.e., Figure 4a–f) and ultra-high-cycle (i.e., Figure 4g–i) fatigue fractures, respectively. Figure 4b,e,h are respectively magnitude views of the area within the red dashed box in Figure 4a,d,g. Figure 4c,f,i are respectively magnitude views of the area within the purple dashed box in Figure 4a,d,h. The yellow dashed line indicates the early initiation and expansion area of fatigue cracks, and the red arrow indicates the specific location of the crack initiation.

In the high cycles, bending fatigue fracture of 316L SS is in line with the common high-cycle fatigue crack initiation characteristics: multiple crack source initiation characteristics, as shown in Figure 4a and d. Another feature of the high-cycle bending fatigue fracture in this test is that cracks originate from the specimen’s upper and lower surfaces simultaneously, as shown in Figure 4d. In the middle of the specimen’s cross-section, two cracks finally intersect and cause fatigue failure, forming this typical fracture feature. It is caused by the gradient distribution of stress along the thickness direction under bending fatigue. As a result, the near-surface stress is the largest, and the surface fatigue crack occurs first.

Figure 4g–i show the fatigue fracture of the ultra-high cycles. It can be seen that, even with the above gradient distribution of stress, the ultra-high cycle bending fatigue crack still initiates as a single crack source, and its contrast is different from the rapid growth zone of the crack in Figure 4g. The fatigue crack propagates from the initiation source area to the other surface of the specimen until failure, also in line with ultra-high cycle fatigue crack initiation characteristics. In Figure 4i, the white dotted line indicates the grain boundary near the crack initiation position. The red dotted line inside the grain shows that the crack expands for a while and pauses for a while because the test is intermittently loading. The white arrow at the edge of the grain boundary refers to secondary cracks caused by the main crack’s expansion. It is a transgranular crack expansion in this grain, and the inside of the grain is relatively smooth.

From the fracture analysis, the fatigue cracks of 316L in the high cycles or very high cycles are all initiated from the surface. The different fractures topography in Figure 4 is due to their different fatigue life, in other words, Figure 4a–f are in the high cycles and Figure 4g–i are in very high cycles. Unlike many structural materials (such as aluminum alloys [31], high-strength steel [8,9,10,11,12,13,14,15,16,17,18,19,20,21,22,23,24,25,26,27,28,29,30,31,32] and titanium alloys [33,34]), from the high cycles to the very high cycles, the fatigue crack initiation site shift from the surface to the inner. The existence of the stress gradient changes the initiation position of the fatigue crack source but does not change the initiation mechanism of the very high cycle fatigue crack.

### 3.2. AFM Observations on the Surface after Fatigue

Although the stress amplitude of ultra-high cycle fatigue is lower than the material’s yield strength, strong plastic deformation still occurs in the very small part of the material, and slip bands are important features of the crystal plastic deformation. The specimen surface is the place of greatest deformation in the above mentioned in very high cycle symmetric bending fatigue tests. Therefore, it is necessary to study the evolution of slip bands on the surface of 316L SS with cycles to understand the nucleation process of bending fatigue cracks. To study the specimen surface used by OM and AFM, it is necessary to repeatedly interrupt the bending fatigue test.

**Figure 5:**
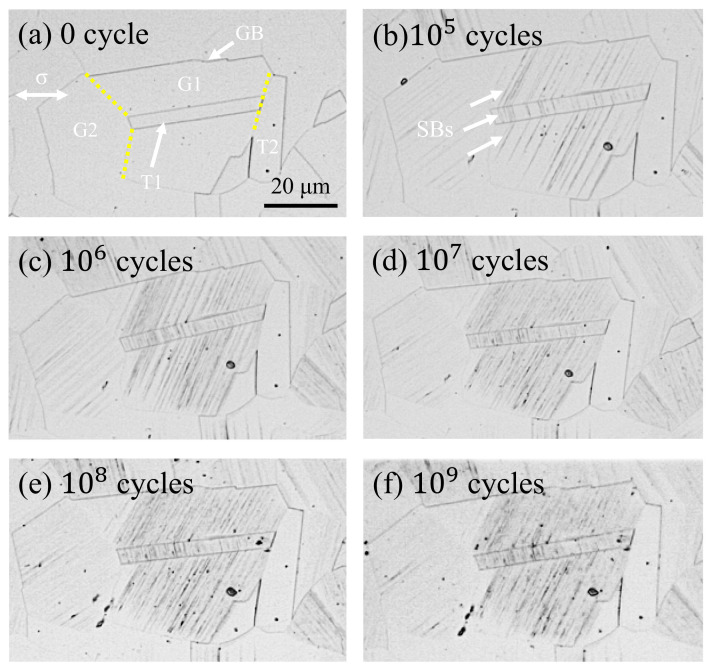
Evolution of slip bands on specimen surface observed by OM. (**a**–**f**) The number of bending cycles is 0, $10^{5}$, $10^{6}$, $10^{7}$, $10^{8}$ and $10^{9}$, respectively. (σ = 322 MPa).

Figure 5 shows the evolution of slip bands in austenite grains on the surface of ultra-high cycle bending fatigue specimens with constant stress amplitude (i.e., σ = 322 MPa). Figure 5a shows the surface of the specimen after electropolishing and corrosion treatment. The scale of all images is the same as shown in Figure 5a. The white arrow refers to the annealed twins, and the yellow dotted line is the grain boundary among G1, G2, T1, and T2. The white arrow in Figure 5b refers to the slip bands in austenite grains after 10^5^ fatigue cycles.

It can be seen from Figure 5a–f that although the specimen surface is subjected to stress, only in certain orientation grains (such as G1 and G2 in Figure 5c) are slip bands formed. Although slip bands are formed in both G1 and G2, the corresponding slip is more intensive in G1. As the cycles increase, the slip band of these grains is denser, especially for G1 in Figure 5b–f. It is consistent with existing research results, that is, the orientation of each grain is divided into soft and hard in materials, and the deformation sequence and amount of deformation of each grain are different, but the deformation of each grain is restricted by its adjacent grains [35]. Secondly, the grains with the highest Schmid factor are more likely to slip under shear stress, which leads to the more prominent slip marks in these grains [18]. Interestingly, even when the fatigue cycles reach 10^9^, there are no slip bands in some grains with individual orientations, indicating that no plastic deformation occurs in these grains, as shown in T2 in Figure 5f.

**Figure 6:**
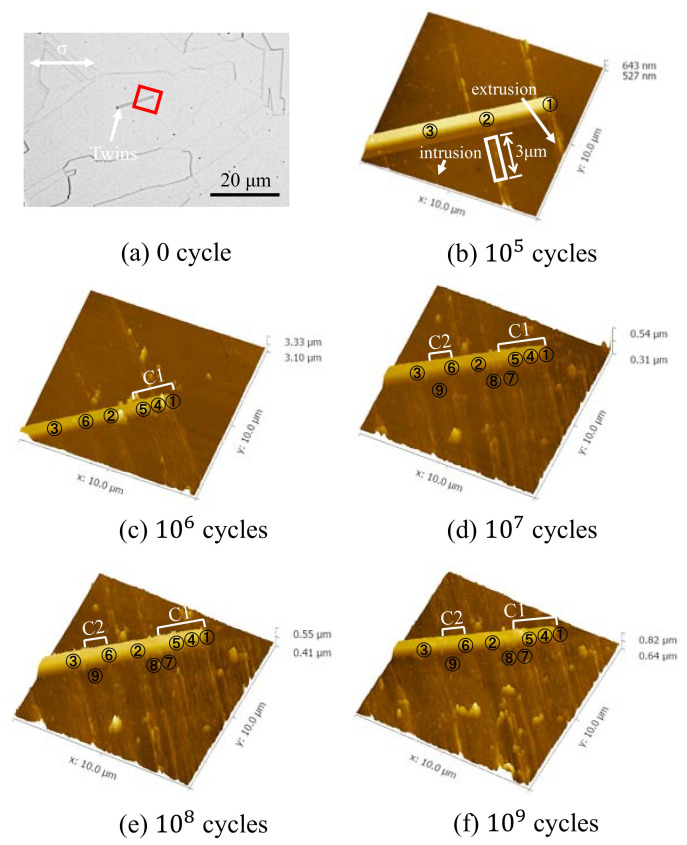
3D morphology of slip bands at different fatigue cycles. (**a**) OM image. (**b**–**f**) AFM images. (σ = 322 MPa).

Figure 6 shows the 3D morphology characteristics of slip bands under different fatigue cycles obtained by AFM at 322 MPa. The scanning region of AFM is the red solid line area in Figure 6a, the size of which is 10 μm × 10 μm, and the white arrow shows annealing twins.

It can be seen from Figure 6b that there are three slip bands in the lower part of annealing twins after $10^{5}$ cycles, named 1, 2, and 3, respectively. Only the region between slip band 1 and 3 is considered. After $10^{6}$ cycles, three new slip bands are added, marked as 4, 5, and 6. When the cycle reaches $10^{7}$, the number of slip bands is 9, marked as 7, 8, and 9. The number of slip bands did not increase until $10^{9}$ cycles. It is well known that the generation of slip bands is often accompanied by the formation of intrusions and extrusions, as shown in Figure 6b. It should be noted that adjacent slip bands are not completely independent growths, that is, slip band 1, 4, and 5 are combined after $10^{6}$ cycles, named C1 in Figure 6c. After $10^{7}$ cycles, slip band 7 also combines with the above three slip bands, as shown in Figure 6c,d. In addition, slip band 6 and 9 also tend to grow together, named C2 in Figure 6d–f. It is observed that intrusions often appear when slip bands are just formed, as shown by slip band 3 and 8 in Figure 6. As the number of cycles increases, some intrusions disappear. The possible reason is that the combined growth of adjacent slip bands causes intrusions between the slip band and the matrix to be squeezed, causing it to disappear gradually.

**Figure 7:**
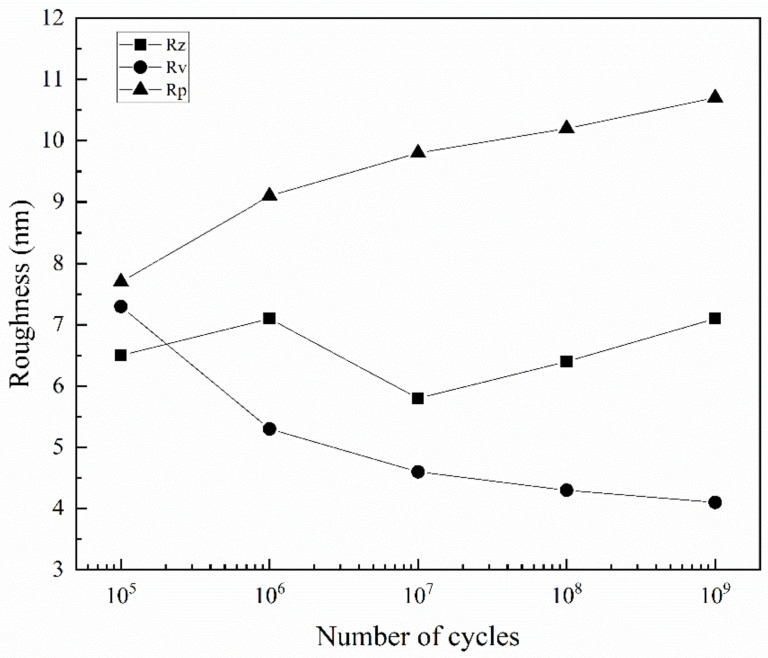
Variation of the roughness of slip band 2. (σ = 322 MPa).

It is easy to measure the contour shape of slip band 2 because it does not grow with other slip bands in the whole fatigue test. Take the 3 μm long section of it near the twin, as shown in the solid white wireframe in Figure 6b, and analyze the variation of the roughness value with the increase of cycles using AFM post-processing software. The result is shown in Figure 7. Among them, Rz is the average maximum height of the roughness, Rv is the maximum roughness valley depth, and Rp is the maximum roughness peak height. Rv and Rp are used to represent the growth of the largest intrusions and extrusions. It can be seen that the overall average maximum roughness has no significant change. The valley depth of the maximum roughness decreases, and the peak height of the maximum roughness increases, with the increase of cycles. However, in the ultra-high cycle fatigue regime, the reduction rate of the maximum roughness valley depth, and the increase rate of the maximum roughness peak height gradually decreases with the increase of fatigue cycles. Already reported in the literature [36], the initial extrusion and intrusion growth rate of slip band are high, and then decreases in the low cycle fatigue regime.

From the above results, it can be concluded that the fatigue limit of 316L SS exists in ultra-high cycles for the following two reasons. At the low-stress amplitude, the extrusion growth rate of the slip band is low, which reduces the accumulation rate of plastic strain in the partial area, delays the initiation time of fatigue cracks, and obtains higher fatigue cycles. Besides, when the sliding motion is not enough to break through the grain boundary, more slip bands are formed in grains, and adjacent slip bands tend to grow together to balance the plastic deformation between grains. The gradual decrease of the maximum roughness valley depth indicates that the merging growth process of slip bands will lead to the disappearance of some intrusions, reduce the stress concentration between the slip band and the matrix to a certain extent, and delay the initiation time of fatigue cracks.

### 3.3. Observation of Surface Fatigue Crack Initiation

**Figure 8:**
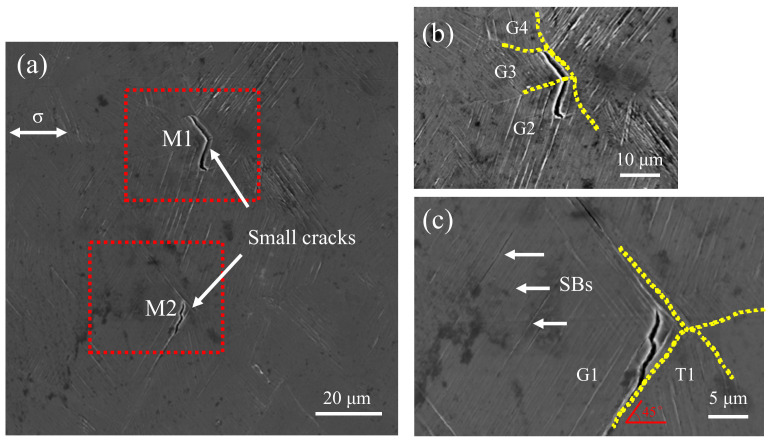
Morphology of small fatigue cracks and slip bands on the specimen surface after fatigue. (**a**) SEM image. (**b**,**c**) The enlarged image of red dashed box in (**a**). (σ = 332 MPa).

Figure 8 shows the free surface morphology of the fatigue failure specimen after $6.09\times 10^{7}$ cycles under SEM. There are two incompletely expanded small fatigue cracks and many significant slip bands in this area. Two small fatigue cracks shown by the white arrow are marked as M1 and M2 in Figure 8a, and slip bands in Figure 8c. The magnitude images circled by the red dashed frame in Figure 8a are shown in Figure 8b and c, respectively. The yellow dashed line refers to the grain boundary in Figure 8b and c. It can be seen that during the fatigue test, the slip band strikes the grain boundary at the grain boundary, making most of the slip motion stop at the grain boundary, showing that the grain boundary hinders the dislocation movement.

It can be seen from Figure 8b and c that both M1 and M2 initiate from the slip band of G2 and G1, respectively. It is consistent with the existing research results, that is, in the field of high cycle fatigue, cracks are preferentially generated from the slip band [11]. It is different from the low cycle fatigue crack initiation mode of this material, which has similar probabilities within the grain and the grain boundary [37]. However, their growth paths are a bit different. It is worth mentioning that the nucleation of M2 is strongly affected by T1.

For M1, it nucleates in the slip band of G2 near the grain boundary, as shown in Figure 8b. When the plastic deformation accumulates enough, it passes through the grain boundary and enters into G3 and propagates along the slip band generated by G3. Next, it expands to the grain boundaries between G3 and G4 and is inhibited and stops growing. The other end of the crack stays in the slip band of G2 and tends to grow across the adjacent slip band under the maximum shear stress. Furthermore, the small fatigue crack deflects from G2 to G3. It is because the plastic deformation is controlled by the shear stress along the active slip plane. As long as the fatigue crack is smaller than some grain sizes, its propagation depends on the active slip system’s direction and usually presents a zigzag pattern on the specimen surface [38].

It can be seen from Figure 8c that M2 is initiated in the slip band of G1 near the twin boundary, which is a common fatigue crack initiation model and is closely related to the orientation of twins and the direction of normal stress. After nucleation, it propagates in a straight line along with the slip band to the twin boundary. However, this straight-line propagation does not continue along with one slip band but forms a step-shaped growth path between the adjacent in the G1. The main growth direction is still along the slip band’s straight-line direction, roughly related to the stress direction is 45°, and the propagation stops at the twin boundary of T1. Besides, the other end of the crack stops at the grain boundary between G1 and G2.

## 4. Discussions

The microstructure has a significant effect on the nucleation and early propagation of fatigue cracks, making the growth of small fatigue cracks in polycrystalline materials very different from that of long cracks [39,40]. It is important to study the relationship between microstructure, deformation mechanism, and the small fatigue crack initiation and its propagation.

In the present study, small fatigue cracks of 316L SS were initiated from the slip band and stopped growing at the grain boundary, as shown in Figure 8b and c. Besides, the nucleation site of M1 and M2 are near the grain boundary and twin boundary, respectively, because these locations have higher stress concentrations, showing a strong correlation with grain orientations and boundaries.

### 4.1. The Role of Slip Bands in the Nucleation and Propagation of Small Cracks

**Figure 9:**
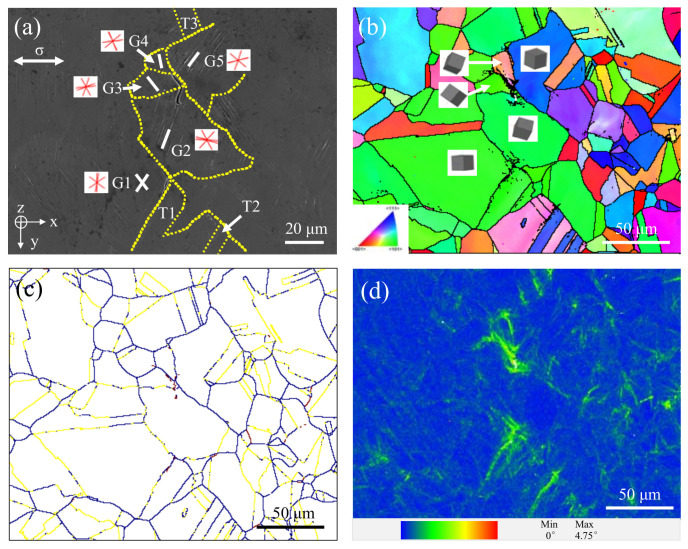
Crystal orientation maps with two small fatigue cracks at 332 MPa: (**a**) SEM image, (**b**) IPF (inverse pole figure) image, (**c**) grain boundaries image (HAGBs (high angle grain boundaries) in blue line, LAGBs (low angle grain boundaries) in purple line, Σ3 twin boundaries in yellow line), and (**d**) LM (local misorientation) image.

The trace planes of all {111} slip planes in G1 to G5 in Figure 9a are marked with red solid line, and the white line is the linear direction of slip bands, and the yellow dotted line is the grain boundary. The corresponding inverse pole figure of the Z-axis and the 3D crystal model with crystal orientation of these grains is shown in Figure 9b. There are 12 slip systems in FCC (face centered cubic) crystals, all the slip planes which are {111} planes, are also called favorable slip planes. Dislocation is the easiest to slip on these planes, so the slip bands in G1–G5 are approximately parallel to the {111} plane, as shown in Figure 9a.

**Figure 10:**
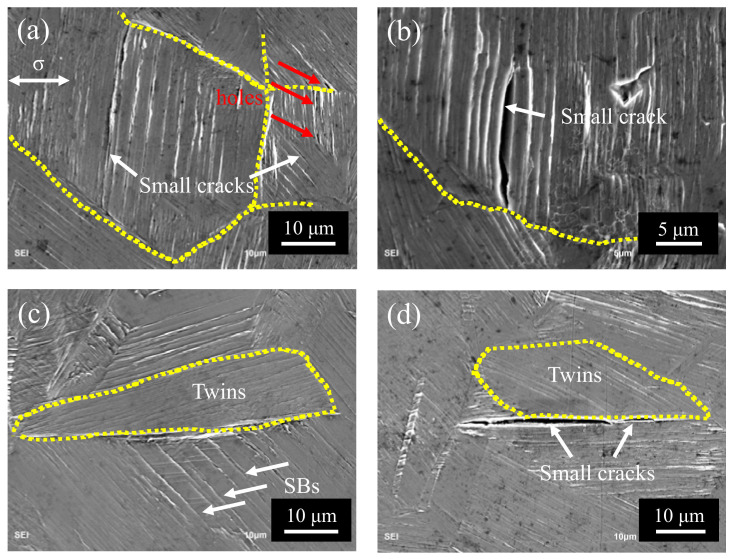
Small cracks in slip bands of the specimen surface at 332 MPa. (**a**,**b**) Small cracks in grains. (**c**,**d**) Small cracks in twin boundaries.

There is literature that the vacuum mode annihilation of dislocations moving forward and backward in the slip band can be regarded as infinitesimal crack propagation [15,16]. With the accumulation of such voids, rows of small holes (as shown in Figure 10a) are gradually formed in the slip band, and these small holes finally merge to form identifiable small cracks in slip bands, as the literature reported for iron [12] and high-Mn TWIP steels (twinning induced plasticity steel) [39], respectively. Moreover, the existing holes in the slip band provide a shortcut for the growth of small fatigue cracks, and it is easy to observe that fatigue cracks tend to grow along the direction of the slip band, as shown in Figure 8b and Figure 10b. After the small fatigue cracks are formed and start to expand, the local area’s stress concentration is reduced, making their expansion discontinuous.

Besides, the path of M1 deviates during its propagation, which is caused by the orientation difference between the favorable slip planes of corresponding grains (i.e., between G2 and G3 as shown in Figure 8b), and the deflection angle is just equal to the angle between the two favorable slip planes and the intersection line of the specimen surface [41].

### 4.2. The Effect of Grain Boundaries in the Nucleation and Propagation of Small Cracks

The LM (local misorientation or named kernel average misorientation, KAM) image in channel 5 of the area where the two small fatigue cracks are located in Figure 9a is shown in Figure 9d, and its value represents the change of local orientation error caused by geometrically necessary dislocation (GND). It is a cloud image drawn by using the average value of orientation deviation between each pixel and its nearest neighbor, showing the general region of defect density increasing, which can be used to study the grain substructure and reflect the strain history of materials [42,43].

It can be seen that there is a maximum LM value in the two small fatigue cracks’ initiation site, and the local plastic strain accumulation is far greater than that in other regions. In addition, the increasing LM value is often observed at the grain boundary, which proves that the grain boundary plays an important role in the initiation of small fatigue cracks. Grain boundaries, especially high angle grain boundaries (HAGBs, see Figure 9c), hinder dislocation movement. Some studies have shown that the impact of slip bands on grain boundaries results in strain incompatibility between grains, causing stress concentration at grain boundaries, making the grain boundaries often act as preferential cracking sites during cyclic deformation [44,45]. According to the experimental results in the literature [37] and this article, the grain boundary as one of the leading factors of small crack initiation has become an important influencing factor, and the probability of fatigue crack initiation caused by twins has changed from small to large from LCF (low cycle fatigue) regime to VHCF regime.

In Figure 9a,d, the LM values in twin boundaries of T2 and T3 are second only to small fatigue crack initiation regions, indicating that considerable plastic strain accumulation is also formed at these locations. It is consistent with many studies, that is, twin boundary is a key factor affecting the mechanical properties of crystal materials [23]. The twin boundary affects the initiation and propagation of the small fatigue crack by changing the plastic deformation accumulation in the local area of the material.

In the present study, twins also play a unique role in the nucleation and propagation of small cracks in 316L SS. One is to make the slip band continue to move through the upper and lower interfaces of twins, thus reducing the stress concentration caused by local strain incompatibility, as shown in Figure 5; it is because the twin boundary can transfer or regulate slip dislocations [46,47]. Secondly, the slip movement inside the grains is blocked, resulting in different slip bands at the upper and lower interfaces of twins, which hinders the slip movement, as shown in Figure 6. Because the twin boundary has the same effect as HAGBs, in other words, inhibiting dislocation motion [48,49], the twin boundary becomes an important position for crack initiation in the VHCF regime, as shown in Figure 8c and Figure 10c,d.

It should be noted that not all twin boundaries are harmful during fatigue cycles. It is shown in Figure 9d that although there is obvious strain accumulation around the twin boundary, which is approximately 45° to the normal stress, only the twin boundary between T1 and G1 eventually forms a small fatigue crack because the twin boundary not only shows the cracking behavior is closely related to its direction [23] but also necessary to consider the grain orientation around it and the size of the twin itself.

## 5. Conclusions

In the present study, the fatigue behavior of 316L SS is investigated using symmetrical bending loading by the very high cycle bending fatigue system developed in the laboratory and examining the slip band features on fatigued surfaces of specimens. The main conclusions can be drawn as follows:

1. Due to the stress gradient of the bending test, fatigue cracks all originate from the surface. At high cycles, fatigue cracks are initiated from multiple crack sources, while at very high cycles, fatigue crack is initiated from a single crack source.
2. Small fatigue cracks tend to initiate at the slip band in the very high cycle bending fatigue regime. Due to the hindrance of the HAGBs to the dislocation movement, the accumulated plastic strain causes the crack initiation site to be near to the grain boundaries (or twin boundaries).
3. The small crack propagation along or across the slip band, approximately 45° to the loading stress direction, is directly influenced by the grain orientations and boundaries.
4. Under low-stress amplitude, failure does not occur because of the growth process of the slip bands. As the cycles increase, the growth rate of the extrusions decreases, and part of the intrusions are squeezed out by the gathering of the slip bands, leading to non-propagating small cracks.

## Figures and Tables

**Table 1:** Mechanical properties of ST 316L SS.

Yield Stress (0.02%; MPa)	Tensile Strength (MPa)	Young’s Modulus (GPa)	Density (kg/m^3^)	Hardness (Hv)
220	580	195	7990	141.1

## References

[1] Molak R., Paradowski K., Brynk T., Ciupinski L., Pakiela Z., Kurzydłowski K.J. (2009). Measurement of mechanical properties in a 316L stainless steel welded joint. Int. J. Press. Vessel. Pip..

[2] Song M., Wang M., Lou X.Y., Rebak R.B., Was G.S. (2019). Radiation damage and irradiation-assisted stress corrosion cracking of additively manufactured 316L stainless steels. J. Nucl. Mater..

[3] Turnbull A., Ryan M.P., Willetts A., Zhou S. (2003). Corrosion and electrochemical behaviour of 316L stainless steel in acetic acid solutions. Corros. Sci..

[4] Naoe T., Xiong Z., Futakawa M. (2016). Gigacycle fatigue behaviour of austenitic stainless steels used for mercury target vessels. J. Nucl. Mater..

[5] Xiong Z., Naoe T., Futakawa M. (2019). Effect of Artificial Defects on the Very High Cycle Fatigue Behavior of 316L Stainless Steel. Metals.

[6] Masaki K., Ochi Y., Matsumura T. (2006). Small crack property of austenitic stainless steel with artificial corrosion pit in long life regime of fatigue. Int. J. Fatigue.

[7] Jiao S., Gao C., Cheng L., Li X., Feng Y. (2016). A Very High-Cycle Fatigue Test and Fatigue Properties of TC17 Titanium Alloy. J. Mater. Eng. Perform..

[8] Wang Q. (2002). Effect of inclusion on subsurface crack initiation and gigacycle fatigue strength. Int. J. Fatigue.

[9] Krupp U. (2007). Fatigue Crack Propagation in Metals and Alloys. Fatigue Crack Propagation in Metals and Alloys.

[10] Szczepanski C., Jha S., Larsen J., Jones J. (2008). Microstructural Influences on Very-High-Cycle Fatigue-Crack Initiation in Ti-6246. Met. Mater. Trans. A.

[11] Mughrabi H. (2015). Microstructural mechanisms of cyclic deformation, fatigue crack initiation and early crack growth. Philos. Trans. R. Soc. A Math. Phys. Eng. Sci..

[12] Wang C., Wagner D., Wang Q., Huang Z., Bathias C. (2015). Very high cycle fatigue crack initiation mechanism according to a 3D model of persistent slip bands formation in α-ferrite. Fatigue Fract. Eng. Mater. Struct..

[13] Chen Y., He C., Yang K., Zhang H., Wang C., Wang Q., Liu Y. (2019). Effects of microstructural inhomogeneities and micro-defects on tensile and very high cycle fatigue behaviors of the friction stir welded ZK60 magnesium alloy joint. Int. J. Fatigue.

[14] He C., Shao X., Yuan S., Fu P., Wu Y., Wang Q., Chen Q. (2019). Small crack initiation and early propagation in an as-extruded Mg-10Gd-3Y-0.5Zr alloy in high cycle fatigue regime. Mater. Sci. Eng. A.

[15] Wilkinson A., Roberts S. (1996). A dislocation model for the two critical stress intensities required for threshold fatigue crack propagation. Scr. Mater..

[16] Krupp U., Alvarez-Armas I. (2014). Short fatigue crack propagation during low-cycle, high cycle and very-high-cycle fatigue of duplex steel—An unified approach. Int. J. Fatigue.

[17] George M., Nziakou Y., Goerke S., Genix A.-C., Bresson B., Roux S., Delacroix H., Halary J.-L., Ciccotti M. (2018). In situ AFM investigation of slow crack propagation mechanisms in a glassy polymer. J. Mech. Phys. Solids.

[18] Man J., Klapetek P., Man O., Weidner† A., Obrtlík K., Polák J. (2009). Extrusions and intrusions in fatigued metals. Part 2. AFM and EBSD study of the early growth of extrusions and intrusions in 316L steel fatigued at room temperature. Philos. Mag..

[19] Man J., Obrtlik K., Polak J. (2009). Extrusions and intrusions in fatigued metals. Part 1. State of the art and history†. Philos. Mag..

[20] Stocker C., Zimmermann M., Christ H.-J. (2011). Localized cyclic deformation and corresponding dislocation arrangements of polycrystalline Ni-base superalloys and pure Nickel in the VHCF regime. Int. J. Fatigue.

[21] Man J., Vystavěl T., Weidner A., Kuběna I., Petrenec M., Kruml T., Polák J. (2012). Study of cyclic strain localization and fatigue crack initiation using FIB technique. Int. J. Fatigue.

[22] Tofique M., Bergström J., Svensson K., Johansson S., Peng R. (2017). ECCI/EBSD and TEM analysis of plastic fatigue damage accumulation responsible for fatigue crack initiation and propagation in VHCF of duplex stainless steels. Int. J. Fatigue.

[23] Zhang Z., Li L., Zhang Z., Zhang P. (2017). Twin boundary: Controllable interface to fatigue cracking. J. Mater. Sci. Technol..

[24] Chai G., Zhou N. (2013). Study of crack initiation or damage in very high cycle fatigue using ultrasonic fatigue test and microstructure analysis. Ultrasonics.

[25] Al Shahrani S., Marrow T.J. (2011). Influence of Twins on Short Fatigue Cracks in Type 316L Stainless Steel. Key Eng. Mater..

[26] Krupp U., Knobbe H., Christ H.-J., Köster P., Fritzen C.-P. (2010). The significance of microstructural barriers during fatigue of a duplex steel in the high- and very-high-cycle-fatigue (HCF/VHCF) regime. Int. J. Fatigue.

[27] Strubbia R., Hereñú S., Giertler A., Alvarez-Armas I., Krupp U. (2014). Experimental characterization of short crack nucleation and growth during cycling in lean duplex stainless steels. Int. J. Fatigue.

[28] Bach J., Möller J.J., Göken M., Bitzek E., Höppel H.W. (2016). On the transition from plastic deformation to crack initiation in the high- and very high-cycle fatigue regimes in plain carbon steels. Int. J. Fatigue.

[29] Zhang J., Li S., Yang Z., Li G., Hui W., Weng Y. (2007). Influence of inclusion size on fatigue behavior of high strength steels in the gigacycle fatigue regime. Int. J. Fatigue.

[30] Grigorescu A., Hilgendorff P.-M., Zimmermann M., Fritzen C.-P., Christ H.-J. (2016). Cyclic deformation behavior of austenitic Cr–Ni-steels in the VHCF regime: Part I—Experimental study. Int. J. Fatigue.

[31] Wang Q., Kawagoishi N., Chen Q. (2006). Fatigue and fracture behaviour of structural Al-alloys up to very long life regimes. Int. J. Fatigue.

[32] Furuya Y., Hirukawa H., Takeuchi E. (2019). Gigacycle fatigue in high strength steels. Sci. Technol. Adv. Mater..

[33] Yang K., He C., Huang Q., Huang Z.Y., Wang C., Wang Q., Liu Y.-J., Zhong B. (2017). Very high cycle fatigue behaviors of a turbine engine blade alloy at various stress ratios. Int. J. Fatigue.

[34] Liu H., Wang H., Huang Z., Wang Q., Chen Q. (2020). Comparative study of very high cycle tensile and torsional fatigue in TC17 titanium alloy. Int. J. Fatigue.

[35] Mineur M., Villechaise P., Méndez J. (2000). Influence of the crystalline texture on the fatigue behavior of a 316L austenitic stainless steel. Mater. Sci. Eng. A.

[36] Polák J. (2003). AFM evidence of surface relief formation and models of fatigue crack nucleation. Int. J. Fatigue.

[37] Mu P., Aubin V., Alvarez-Armas I., Armas A. (2013). Influence of the crystalline orientations on microcrack initiation in low-cycle fatigue. Mater. Sci. Eng. A.

[38] Sistaninia M., Niffenegger M. (2015). Fatigue crack initiation and crystallographic growth in 316L stainless steel. Int. J. Fatigue.

[39] Hamada A., Karjalainen P., Puustinen J. (2009). Fatigue behavior of high-Mn TWIP steels. Mater. Sci. Eng. A.

[40] He C., Wu Y., Peng L., Su N., Chen Q., Yuan S., Liu Y., Wang Q. (2019). Effect of microstructure on small fatigue crack initiation and early propagation behavior in Mg-10Gd-3Y-0.3Zr alloy. Int. J. Fatigue.

[41] Zhai T., Wilkinson A., Martin J. (2000). A crystallographic mechanism for fatigue crack propagation through grain boundaries. Acta Mater..

[42] Kamaya M., Kubushiro K., Sakakibara Y., Suzuki S., Morita H., Yoda R., Kobayashi D., Yamagiwa K., Nishioka T., Yamazaki Y. (2016). Round robin crystal orientation measurement using EBSD for damage assessment. Mech. Eng. J..

[43] Gussev M., Leonard K. (2019). In situ SEM-EBSD analysis of plastic deformation mechanisms in neutron-irradiated austenitic steel. J. Nucl. Mater..

[44] Kim W., Laird C. (1978). Crack nucleation and stage I propagation in high strain fatigue—II. mechanism. Acta Met..

[45] Zhang Z., Wang Z. (2008). Grain boundary effects on cyclic deformation and fatigue damage. Prog. Mater. Sci..

[46] Bufford D., Liu Y., Wang J., Wang H., Zhang X. (2014). In situ nanoindentation study on plasticity and work hardening in aluminium with incoherent twin boundaries. Nat. Commun..

[47] Wang W., Liu T., Cao X., Lu Y., Shoji T. (2017). In-situ observation on twin boundary evolution and crack initiation behavior during tensile test on 316L austenitic stainless steel. Mater. Charact..

[48] Rémy L. (1981). The interaction between slip and twinning systems and the influence of twinning on the mechanical behavior of fcc metals and alloys. Met. Mater. Trans. A.

[49] Barbier D., Gey N., Allain S., Bozzolo N., Humbert M. (2009). Analysis of the tensile behavior of a TWIP steel based on the texture and microstructure evolutions. Mater. Sci. Eng. A.

